# Composite Al_2_O_3_-Ce:LuAG Phosphor Ceramics with High Luminous Efficacy and Thermal Conductivity for High-Brightness Laser Lighting

**DOI:** 10.3390/ma19183811

**Published:** 2026-09-08

**Authors:** Haiming Li, Ziqiu Cheng, Zhenzhen Zhou, Chen Hu, Junhao Ye, Dong Huang, Yanbin Wang, Tingsong Li, Heng Liu, Shisheng Lin, Denis Yu. Kosyanov, Daqin Chen, Duyou Lu, Jiang Li

**Affiliations:** 1 State Key Laboratory of High Performance Ceramics, Shanghai Institute of Ceramics, Chinese Academy of Sciences, Shanghai 201899, China; lhm1981007047@163.com (H.L.); chengziqiu20@mails.ucas.ac.cn (Z.C.); zhouzhenzhen@mail.sic.ac.cn (Z.Z.); huchen@mail.sic.ac.cn (C.H.); yejunhao22@mails.ucas.ac.cn (J.Y.); huangdong@mail.sic.ac.cn (D.H.); wangyanbin@mail.sic.ac.cn (Y.W.); litingsong@mail.sic.ac.cn (T.L.); 17537722500@163.com (H.L.); 2 College of Materials Science and Engineering, Nanjing Tech University, Nanjing 211816, China; 3 Center of Materials Science and Optoelectronics Engineering, University of Chinese Academy of Sciences, Beijing 100049, China; 4 College of Physics and Energy, Fujian Normal University, Fuzhou 350117, China; linshisheng@fjnu.edu.cn (S.L.); dqchen@fjnu.edu.cn (D.C.); 5 Fujian Provincial Collaborative Innovation Center for Advanced High-Field Superconducting Materials and Engineering, Fujian Normal University, Fuzhou 350117, China; 6 Polytechnic Institute Vladivostok, Far Eastern Federal University, 690922 Vladivostok, Russia; kosianov.diu@dvfu.ru; 7 Institute of Automation and Control Processes, Far Eastern Branch of the Russian Academy of Sciences, 690041 Vladivostok, Russia

**Keywords:** Al_2_O_3_-Ce:LuAG, composite phosphor ceramics, luminescence properties, thermal conductivity, laser lighting

## Abstract

**Highlights:**

- High sintering activity Al_2_O_3_-Ce:LuAG nanopowders were prepared via a co-precipitation method.
- Ceramics vacuum-sintered at 1750 °C for 10 h exhibited excellent thermal stability (I_450K_/I_RT_ = 96%).
- The thermal conductivity of the “1750 °C × 10 h” sample was 15.6 W·m^−1^·K^−1^ at room temperature.
- Al_2_O_3_ particles are uniformly dispersed in the Ce:LuAG matrix.
- Luminous efficacy reached 286 lm/W, with a saturation threshold at 18 W·mm^−2^.

**Abstract:**

Despite the success of using Al_2_O_3_ as a secondary phase in Ce:LuAG phosphor ceramics (PCs), there is still room for improvement in the compositional design of biphasic PCs, as well as in their luminescent and thermal performance. In this study, nanopowders with 40 wt.% Al_2_O_3_-0.4at.% Ce:LuAG stoichiometry were synthesized via a co-precipitation approach. Subsequently, a series of compositionally uniform PCs was successfully fabricated by adjusting the vacuum sintering temperature and dwelling time. The grain size distributions of the Al_2_O_3_ and LuAG phases, as well as the evolution of porosity and pore size, were systematically analyzed and correlated with the sintering conditions. The addition of Al_2_O_3_ has been demonstrated to enhance the thermal properties of ceramics. The thermal conductivity of the “1750 °C × 10 h” sample was 15.6 W·m^−1^·K^−1^ at room temperature. Concurrently, it exhibited excellent thermal quenching behavior, retaining 96% of its luminescence intensity upon heating to 450 K. Its fluorescence lifetime was determined to be 21.06 ns. Under 450 nm laser excitation, the optimized PC attained a luminous efficacy of 286 lm·W^−1^ at 1 W·mm^−2^. In addition, the luminous flux increased continuously with laser power from 1 to 20 W·mm^−2^ without any sign of saturation, reaching a maximum of 2500 lm. The findings indicate that biphasic 40 wt.% Al_2_O_3_-0.4at.% Ce:LuAG PCs have potential as high-flux, green-color converters for next-generation high-power laser lighting. Furthermore, a laser illumination prototype device incorporating 40 wt.% Al_2_O_3_-0.4at.% Ce:LuAG ceramic samples and a 10 W blue laser was constructed. This device emits white light with an illumination range exceeding 500 m, thereby demonstrating its potential applications in laser-driven lighting.

## 1. Introduction

The rapid advancement of solid-state lighting technology, marked by its increasing power density and brightness, has led to the imposition of elevated performance requirements on light-conversion materials [1,2,3,4,5]. The selection of LuAG (Lu_3_Al_5_O_12_) as the core material for laser lighting systems is primarily due to its inherent advantages [6,7]. Firstly, it exhibits high crystal structural stability, enabling it to withstand high-density laser irradiation [8,9,10,11,12,13]. Secondly, the LuAG matrix is doped with Ce^3+^, producing green emission at 525–527 nm, which aligns with the spectral composition required for white light illumination. However, the LuAG matrix also has significant limitations: its intrinsic thermal conductivity is only approximately 8 W·m^−1^·K^−1^, far below the thermal management requirements of laser lighting systems; simultaneously, under high-power conditions, Ce:LuAG luminescent ceramics exhibit significant emission saturation, leading to a decrease in light output efficiency [14,15,16,17,18,19]. More crucially, under conditions of high-power laser excitation, the color coordinates of single-phase Ce:LuAG ceramics undergo significant shifts due to thermal accumulation effects, thereby compromising the quality of the lighting [20,21,22,23].

In addressing the issue of inadequate thermal conductivity within the LuAG matrix, researchers directed their attention toward aluminum oxide (Al_2_O_3_) as a second-phase additive [24,25,26,27]. This choice stems not only from Al_2_O_3_’s excellent thermal properties but also from its ability to simultaneously resolve two core challenges facing light-conversion materials: light-scattering efficiency and thermal management capability. From the perspective of light scattering, Al_2_O_3_ nanoparticles function as effective light-scattering centers, thereby significantly enhancing the uniformity of incident blue laser distribution within the ceramic [28,29,30,31,32]. In regard to the issue of thermal management, the high thermal conductivity of Al_2_O_3_ (>30 W·m^−1^·K^−1^) effectively compensates for the inadequate thermal conductivity of the LuAG matrix. The Al_2_O_3_-Ce:LuAG composite phosphor ceramics (CPCs) system signifies a substantial advancement in the domain of high-power solid-state lighting materials [33,34]. This system effectively addresses the fundamental challenges encountered by conventional single-phase phosphor materials, including inadequate thermal stability and constrained luminous efficiency [35,36,37,38].

In recent years, research on Al_2_O_3_-Ce:LuAG composite phosphor ceramics (CPCs) [39,40] has made significant progress. Zhang et al. demonstrated that these composite ceramics exhibit high LF density in high-brightness displays [41]. Cheng et al. successfully prepared green Al_2_O_3_-Ce:LuAG CPCs using the solid-state reaction sintering technology [42]. This development served to enhance the thermal stability and operational performance of ceramic phosphor [43] converters under high-power laser irradiation [44]. Subsequently, to overcome compositional inhomogeneity in multiphase PCs prepared by reaction sintering, Wang et al. employed a co-precipitation route to enhance the powder uniformity and sinterability [45]. It was established that increasing the Al^3+^ content in the mixed metal salt solution of Al^3+^, Lu^3+^, and Ce^3+^, enabled the preparation of biphasic Al_2_O_3_-Ce:LuAG PCs with excellent compositional and luminescent uniformity. However, there is still room for improvement in the compositional design of multiphase PCs, as well as in their luminescence and thermal performance.

In this work, 40 wt.% Al_2_O_3_-0.4at.% Ce:LuAG nanopowders were synthesized via co-precipitation followed by air calcination. Subsequently, a series of compositionally uniform PCs was successfully fabricated by adjusting the vacuum sintering temperature and dwelling time. The phase composition, microstructure, and optical and luminescent properties of the Al_2_O_3_-Ce:LuAG ceramics were investigated.

## 2. Materials and Methods

The 40 wt.% Al_2_O_3_-0.4at.%Ce:LuAG nanopowders were synthesized via the co-precipitation method. The weight fraction of the corundum phase corresponds to an excess of Al^3+^ (in terms of Ce:LuAG stoichiometry) and Al_2_O_3_ in the entire mixture of 58 and 85 mol.%, respectively. Lu(NO_3_)_3_ and Ce(NO_3_)_3_ solutions were prepared by dissolving Lu_2_O_3_ (99.99%, Shanghai Jingyun Material Technology Co., Ltd., Shanghai, China) and CeO_2_ (99.999%, Changting Golden Dragon Rare-Earth Co., Ltd., Longyan, China) in hot high-purity nitric acid. An Al(NO_3_)_3_ solution was prepared by dissolving Al(NO_3_)_3_·9H_2_O (99.0%, Sinopharm Chemical Reagent Co., Ltd., Shanghai, China) in deionized water. The metal nitrate solutions were mixed and diluted to 0.5 M. The precipitant solution was obtained by dissolving ammonium hydrogen carbonate (AHC) (analytical grade, Aladdin, Shanghai, China) in deionized water and diluting to 1.5 M. Ammonium sulfate (99.0%, Sinopharm Chemical Reagent Co., Ltd., Shanghai, China) was added to the precipitant as a dispersant. Reverse-strike titration was then performed by dripping the mixed metal nitrates into the AHC at a rate of 20 mL/min at room temperature (RT). After aging for 30 min, the resulting suspension was washed three times with deionized water and twice with absolute ethanol using repeated dispersion and centrifugation. The precursor was dried at 70 °C for 48 h, sieved through a 200-mesh screen, and calcined at 1100 °C for 4 h. The obtained powders were dry-pressed into pellets and vacuum-sintered at 1700, 1725, and 1750 °C for 3 h, as well as at 1750 °C for 10 h. Finally, the composite ceramics were post-annealed in air at 1450 °C for 10 h. For further studies, the ceramic samples were mirror-polished on both sides to a thickness of 1 mm. According to the sintering conditions described above, the CPCs were labeled Samples 1–4.

Phase identification was carried out by X-ray diffraction (XRD; Ultima IV, Rigaku Corp., Tokyo, Japan) in the 2θ range of 15–75° using nickel-filtered Cu Kα radiation. The microstructures of the powders and thermally etched ceramic surfaces were examined by a field-emission scanning electron microscopy (FESEM; SU8220, Hitachi, Ltd., Tokyo, Japan, and Sigma300, Carl Zeiss, Oberkochen, Germany). The porosity of the Al_2_O_3_-Ce:LuAG CPCs was calculated from density data measured using the Archimedes principle. The theoretical full density of 40 wt.% Al_2_O_3_-0.4at.%Ce:LuAG biphasic ceramic is 5.25 g/cm^3^ (obtained according to the rule of mixtures). The average grain sizes were measured by the common linear intercept analysis (more than 200 grains were counted) according to the equation GS = 1.56 L, where L is the mean intercept. The total transmittance over the 250 to 800 nm wavelength range was tested by a UV-VIS-NIR spectrophotometer (Cary 5000, Varian Medical Systems, Inc., Palo Alto, CA, USA). UV-VIS fluorescence spectra were recorded using a homemade multifunctional combined fluorescence spectrum test system (SicOmni-I). The system was equipped with a VX-XBO 150 W xenon lamp, an Omni-λ3007 monochromator (Zolix, Beijing, China), and a PMTH-S1-CR131 photomultiplier tube. The spectral wavelength was calibrated using an LHM254 mercury lamp. Variable-temperature photoluminescence (PL) spectra were measured by an F-4600 spectrofluorometer (Hitachi, Tokyo, Japan). The luminescence performance under 450 nm laser excitation was examined in reflection mode. The related luminous efficacy (LE), luminous flux (LF), and electroluminescent properties at RT were measured using an integrating sphere connected to a CCD OHSP-350 spectrometer (Hangzhou Hopoo Light and Color Technology Co., Ltd., Hangzhou, China).

## 3. Results and Discussion

**Figure 1:**
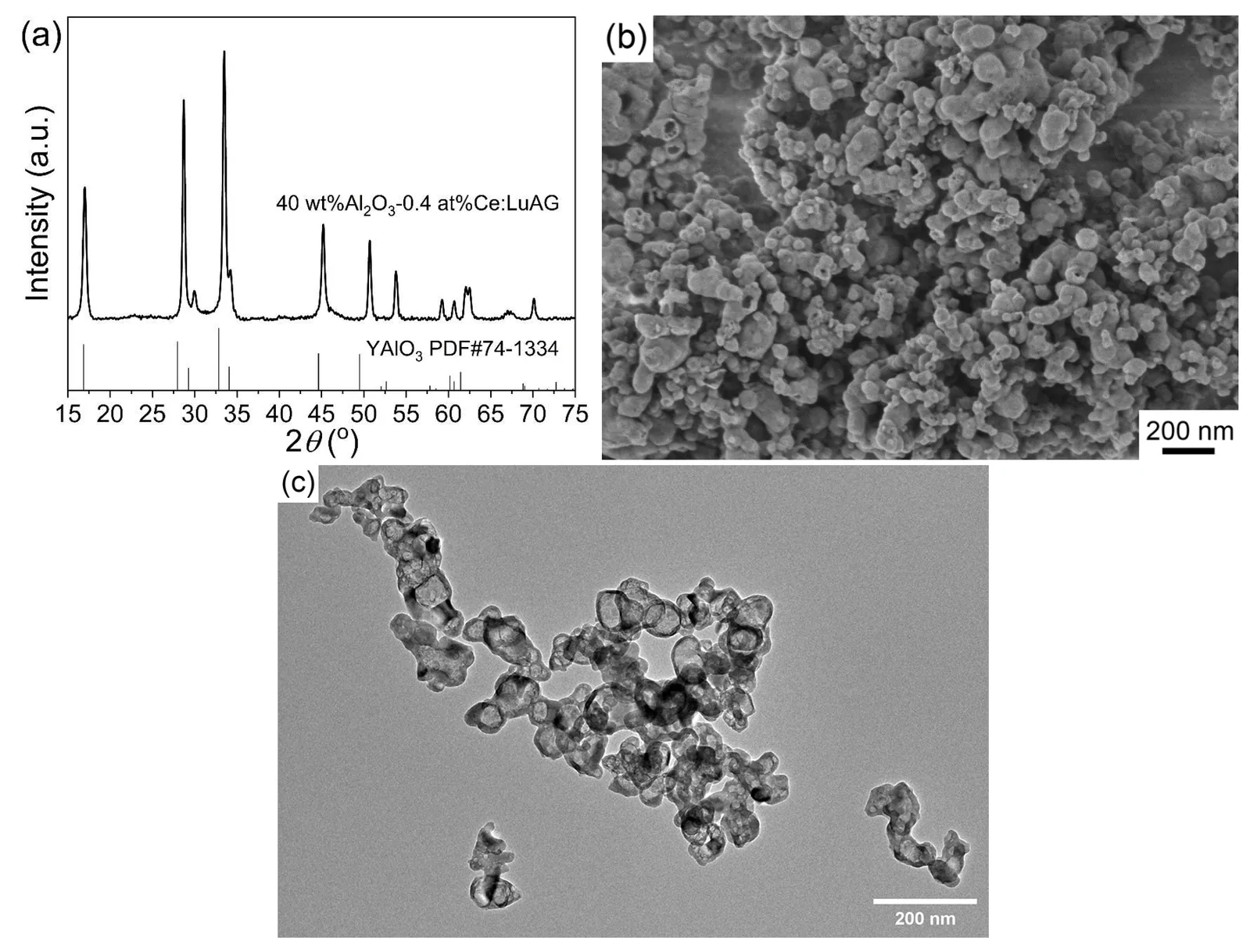
(**a**) XRD pattern, (**b**) FESEM and (**c**) TEM micrographs of Al_2_O_3_-Ce:LuAG nanopowders.

Figure 1 shows the XRD pattern, FESEM and TEM micrographs of nanopowders with 40 wt.% Al_2_O_3_-0.4at.%Ce:LuAG stoichiometry after air calcination at 1100 °C for 4 h. According to XRD results, nanopowders show a single-phase perovskite structure indexed to YAP (YAlO_3_, PDF#74-1334), which serves as a structural analog for LuAP in the absence of a standard database PDF card for LuAP. This phase evolution is attributed to the significant excess of Al^3+^ ions relative to Lu^3+^ in LuAG, which alters the local stoichiometry and promotes the formation of the perovskite LuAP phase. The atomic-scale homogeneity achieved via co-precipitation promotes this phase transformation, as the well-dispersed Al^3+^ and Lu^3+^ precursors facilitate perovskite nucleation at intermediate temperatures. This observation is in agreement with previous reports showing that LuAG can convert to LuAP under Al^3+^-rich conditions [45]. LuAP may be considered a thermodynamically stable intermediate phase in the Lu_2_O_3_-Al_2_O_3_ system at 1100 °C, whereas elevated temperatures or stoichiometrically balanced conditions promote its transformation toward the garnet phase. The nanopowders were composed of aggregates formed by interconnected, irregularly shaped primary particles with an average size of about 100 nm (Figure 1b,c).

**Figure 2:**
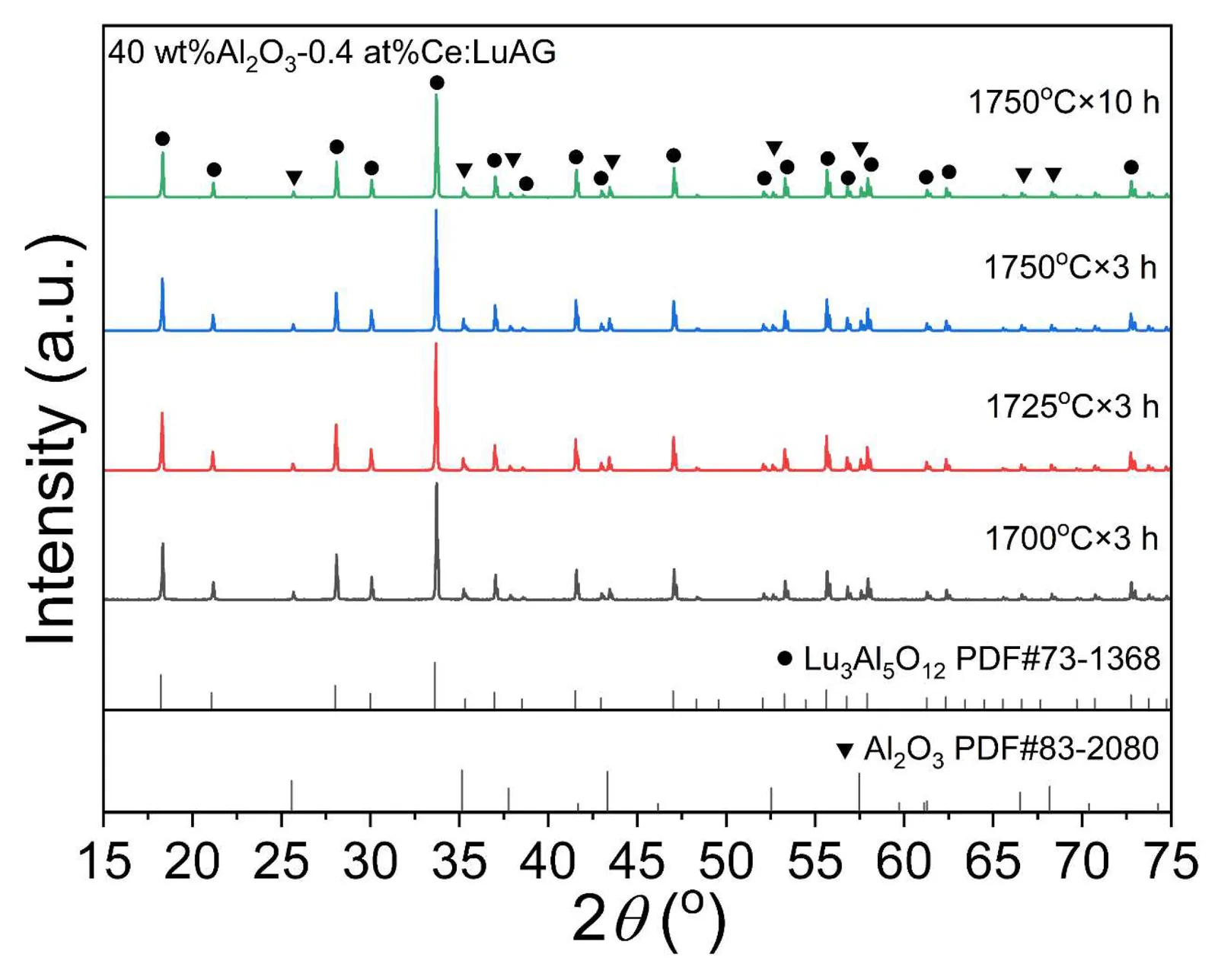
XRD patterns of Al_2_O_3_-Ce:LuAG CPCs.

The XRD patterns of CPCs are shown in Figure 2. All samples in the series consisted of garnet LuAG (PDF # 73-1368) and corundum Al_2_O_3_ (PDF # 83-2080) phases, and no additional phases were detected. This result indicates the complete LuAP→LuAG phase transformation under high-temperature sintering conditions, resulting in biphasic Al_2_O_3_-Ce:LuAG ceramics.

**Figure 3:**
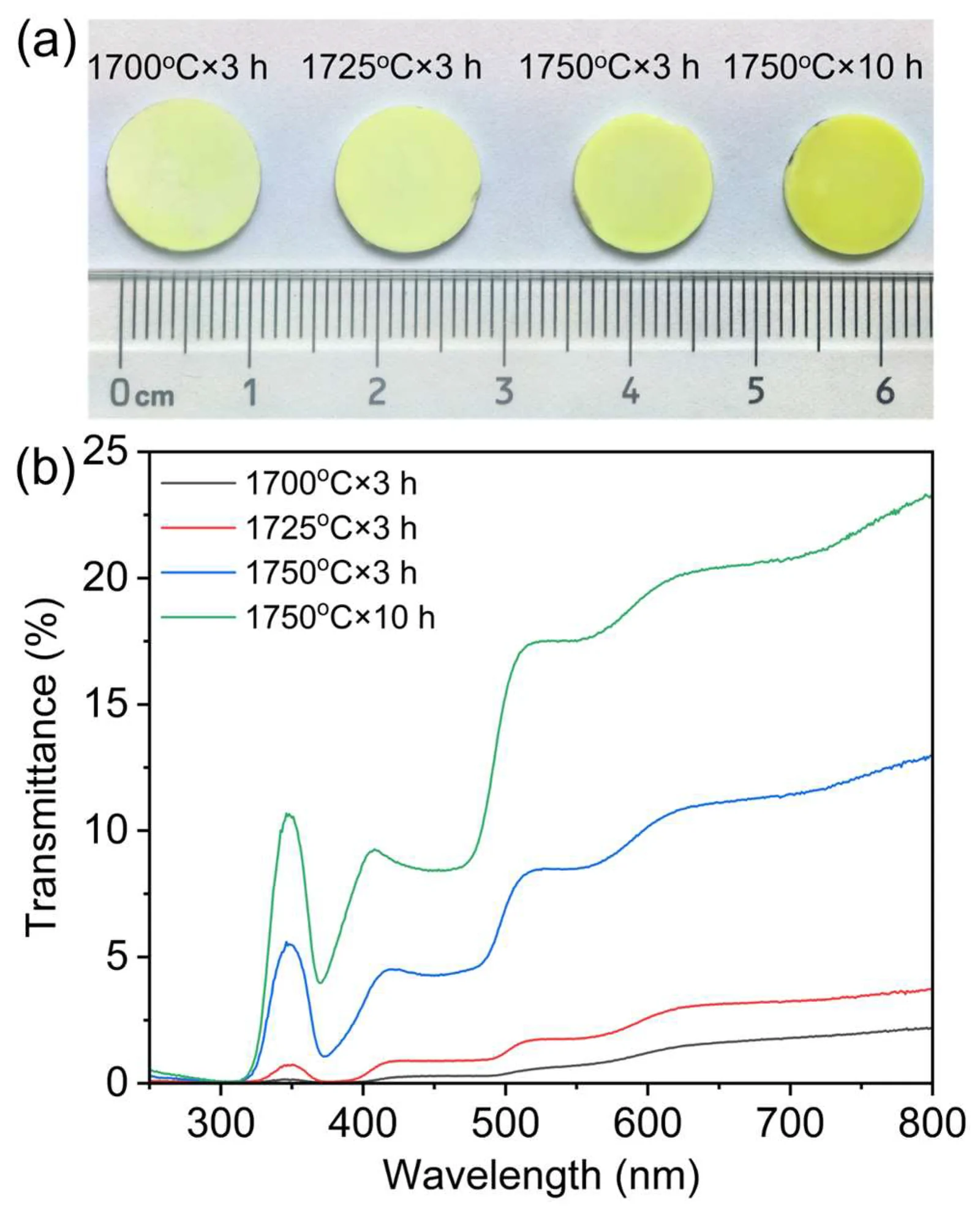
(**a**) Photograph and (**b**) total transmittance spectra of Al_2_O_3_-Ce:LuAG CPCs (1 mm thickness) vacuum-sintered under different temperature–time conditions.

As illustrated in Figure 3a, a photograph of Al_2_O_3_-Ce:LuAG CPCs that were vacuum-sintered at varying temperatures for differing lengths of time is presented. The color of ceramic specimens gradually brightens in accordance with the temperature and duration of the vacuum sintering process. Figure 3b shows the total transmittance spectra of post-annealed Al_2_O_3_-Ce:LuAG CPCs with a thickness of 1 mm, which were vacuum-sintered under different temperature–time conditions. As the vacuum sintering temperature and holding time increase, the transmittance of the ceramic also gradually increases. A maximum value of 23% at 800 nm was achieved for the sample sintered at 1750 °C for 10 h. The absorption peaks at 360 and 460 nm in all CPCs correspond to the 4f→5d_2_ and 4f→5d_1_ transitions of Ce^3+^, respectively.

**Figure 4:**
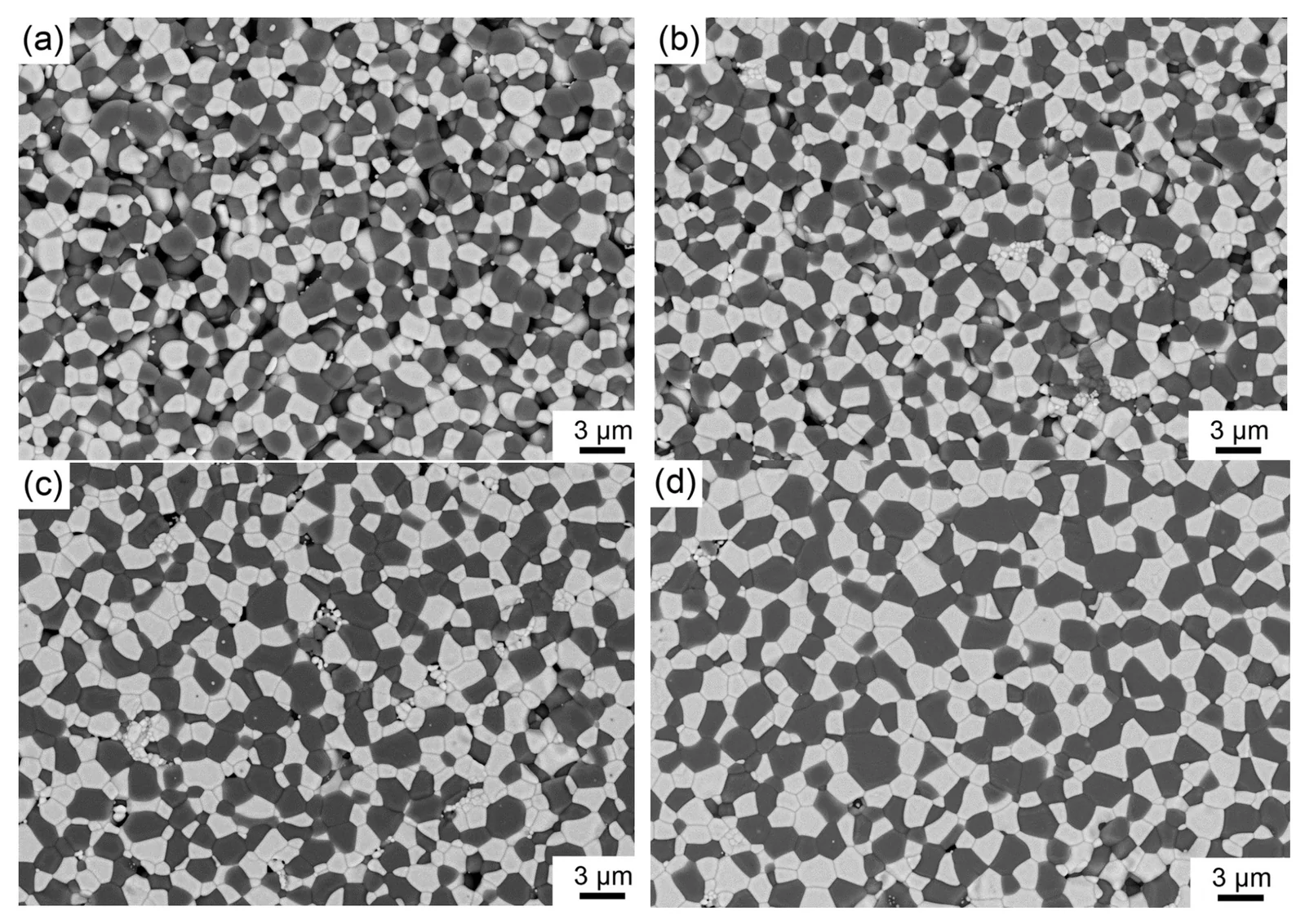
FESEM micrographs of Al_2_O_3_-Ce:LuAG CPCs vacuum-sintered under different temperature–time conditions: (**a**) 1700 °C × 3 h, (**b**) 1725 °C × 3 h, (**c**) 1750 °C × 3 h, and (**d**) 1750 °C × 10 h.

Figure 4 shows the FESEM micrographs of Al_2_O_3_-Ce:LuAG CPCs. According to the contrast in backscattered electron images, the bright and dark grains correspond to Ce:LuAG and Al_2_O_3_, respectively. In composites, the Al_2_O_3_ particles are uniformly dispersed in the Ce:LuAG matrix. As the sintering temperature and time increase, the alumina phase gradually evolves from a dispersed to a continuous phase. It is known that the pore structure has a direct influence on the trajectory of incident blue light, which in turn affects the absorption efficiency and thus the LE of phosphors. The relative density of the CPCs increases with the increase in sintering temperature and time, reaching 79%, 91%, 99%, and 100%, respectively. This trend is consistent with the optical transmission behavior presented in Figure 3b.

**Figure 5:**
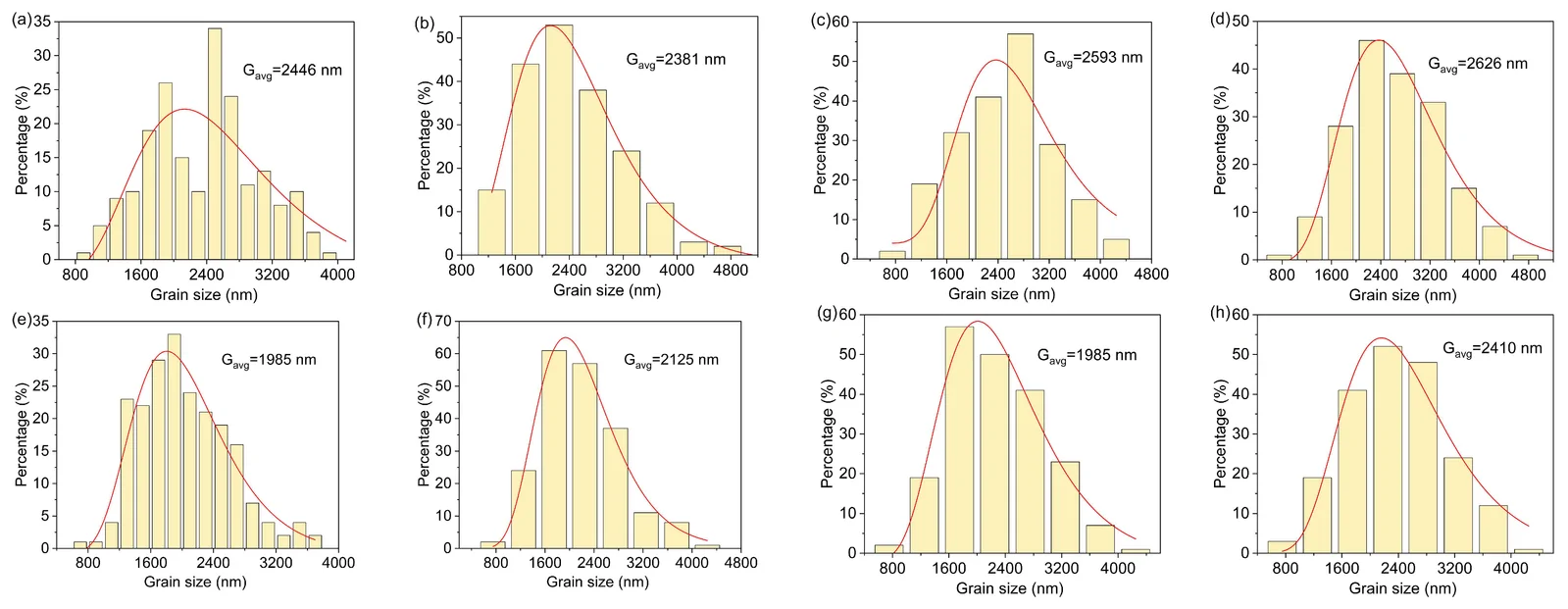
Grain size distribution histograms of Al_2_O_3_ (**a**–**d**) and LuAG (**e**–**h**) phases in Al_2_O_3_-Ce:LuAG CPCs vacuum-sintered under different conditions: (**a**,**e**) 1700 °C × 3 h; (**b**,**f**) 1725 °C × 3 h; (**c**,**g**) 1750 °C × 3 h; (**d**,**h**) 1750 °C × 10 h.

Figure 5 shows the Grain size distribution histograms of Al_2_O_3_ and LuAG phases in Al_2_O_3_-Ce:LuAG CPCs vacuum-sintered under different conditions. The grain sizes were measured from SEM micrographs of polished and thermally etched surfaces. According to the contrast in backscattered electron images, the bright and dark grains correspond to Ce:LuAG and Al_2_O_3_, respectively. The results show that, with increasing sintering temperature and holding time, the average grain size of the Al_2_O_3_ phase increases from 1511 nm to 1557 nm, 1612 nm, and 1702 nm, respectively. Similarly, the average grain size of the LuAG phase increases from 1303 nm to 1389 nm, 1464 nm, and 1539 nm, respectively. This trend indicates that higher sintering temperature and longer holding time promote grain growth in both phases.

**Figure 6:**
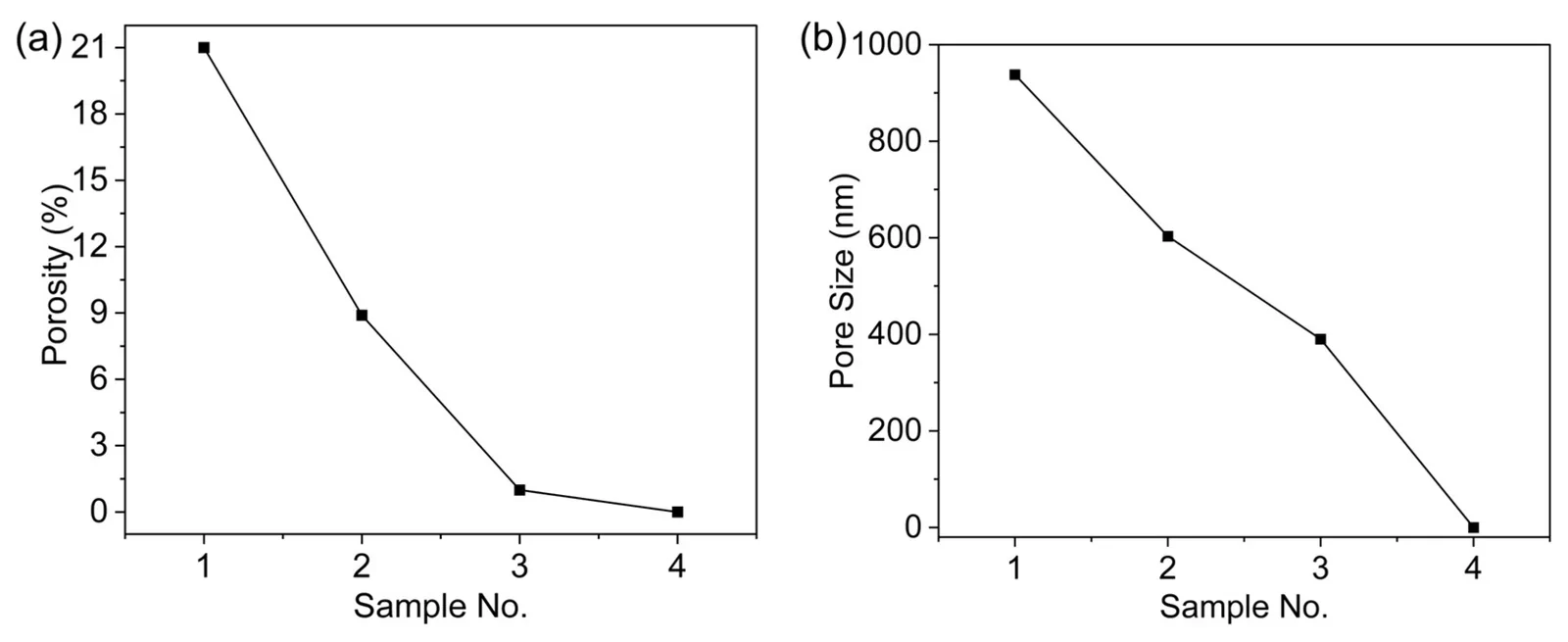
(**a**) Porosity and (**b**) pore size of Al_2_O_3_-Ce:LuAG CPCs sintered at different temperatures and time.

Figure 6 shows the porosity and pore size of Al_2_O_3_-Ce:LuAG CPCs sintered at different temperatures and time. The results show that the porosity decreases monotonically with increasing sintering temperature and holding time. Correspondingly, the average pore size decreases from about 938 nm to nearly zero, indicating that the ceramics become almost fully dense under the optimized sintering conditions. This behavior can be attributed to the progressive densification process: as sintering proceeds, pores are gradually eliminated through grain boundary diffusion and pore migration, leading to a simultaneous reduction in both porosity and pore size.

**Figure 7:**
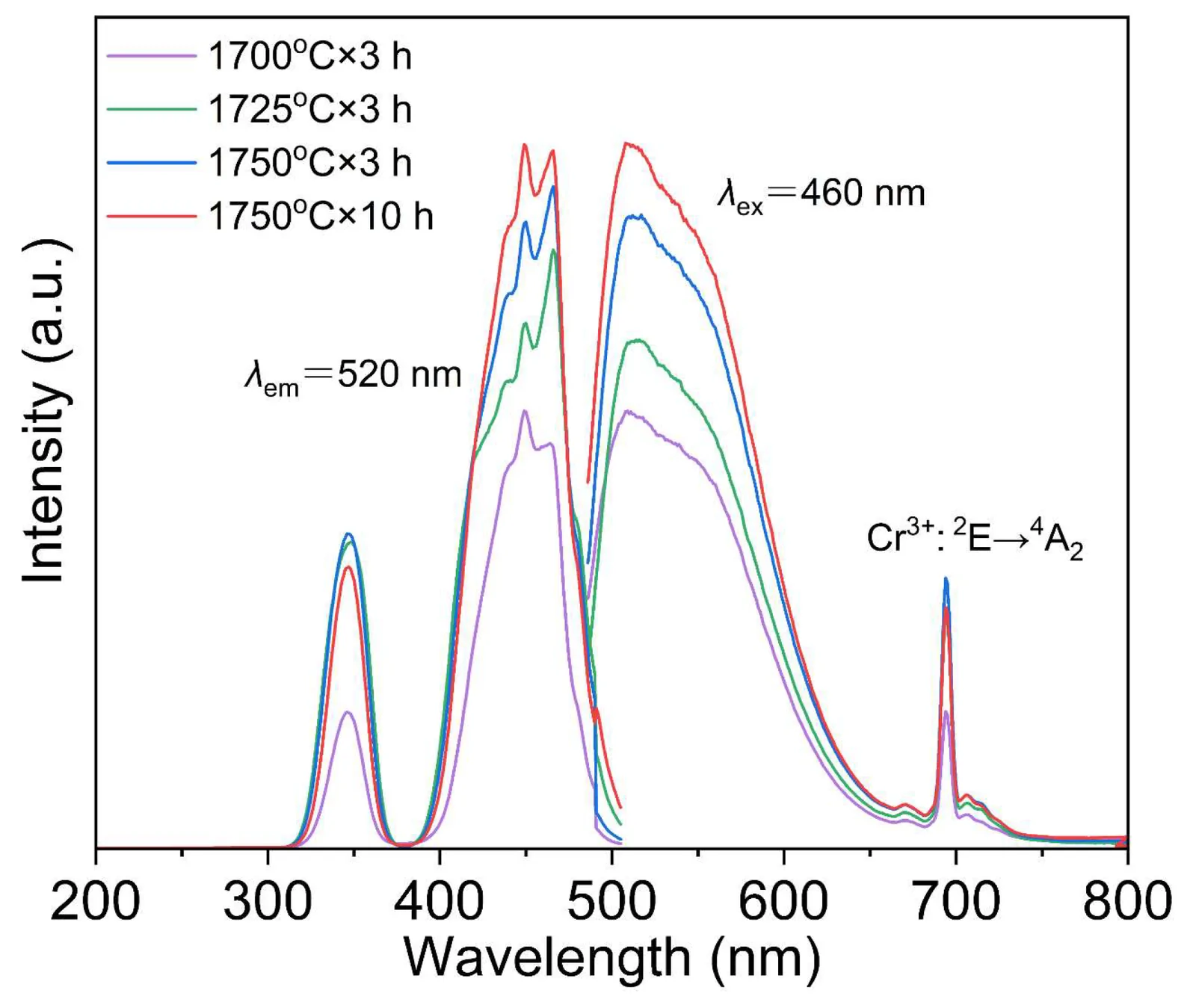
PL/PLE spectra of Al_2_O_3_-Ce:LuAG CPCs vacuum-sintered under different temperature–time conditions.

Figure 7 shows the excitation and emission spectra of CPCs at RT. The excitation peaks in the 300–400 nm and 400–500 nm regions are attributed to the transitions of Ce^3+^ from the ground state ^2^*F*_5/2_ to the two excited states 5*d*_2_ and 5*d*_1_, respectively. Under 469 nm excitation, the emission band in the 500–650 nm range originates from the 4*f*^0^5*d*^1^ → 4*f*^1^5*d*^0^ transition. With the increase in sintering temperature and time, the excitation and emission peak intensities exhibit a gradual upward trend. The internal quantum efficacy η(IQE) of the ceramic sample vacuum-sintered at 1750 °C for 10 h was measured to be 61.3%. Furthermore, the emission peak at 695 nm may be attributed to the ^2^*E* → ^4^*A*_2_ transition of Cr^3+^, potentially introduced from the aluminum nitrate solution due to the relatively low purity of the Al(NO_3_)_3_·9H_2_O raw materials (99.0%).

**Figure 8:**
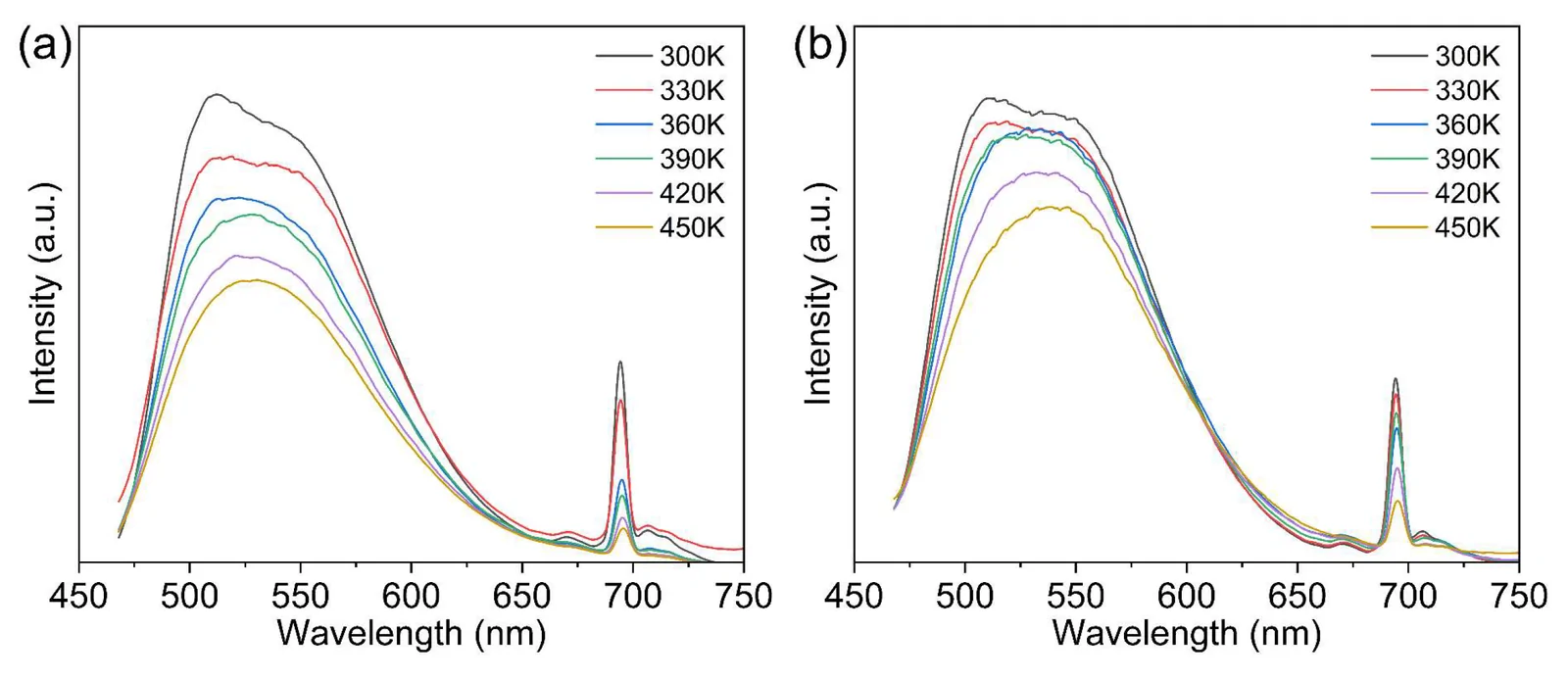

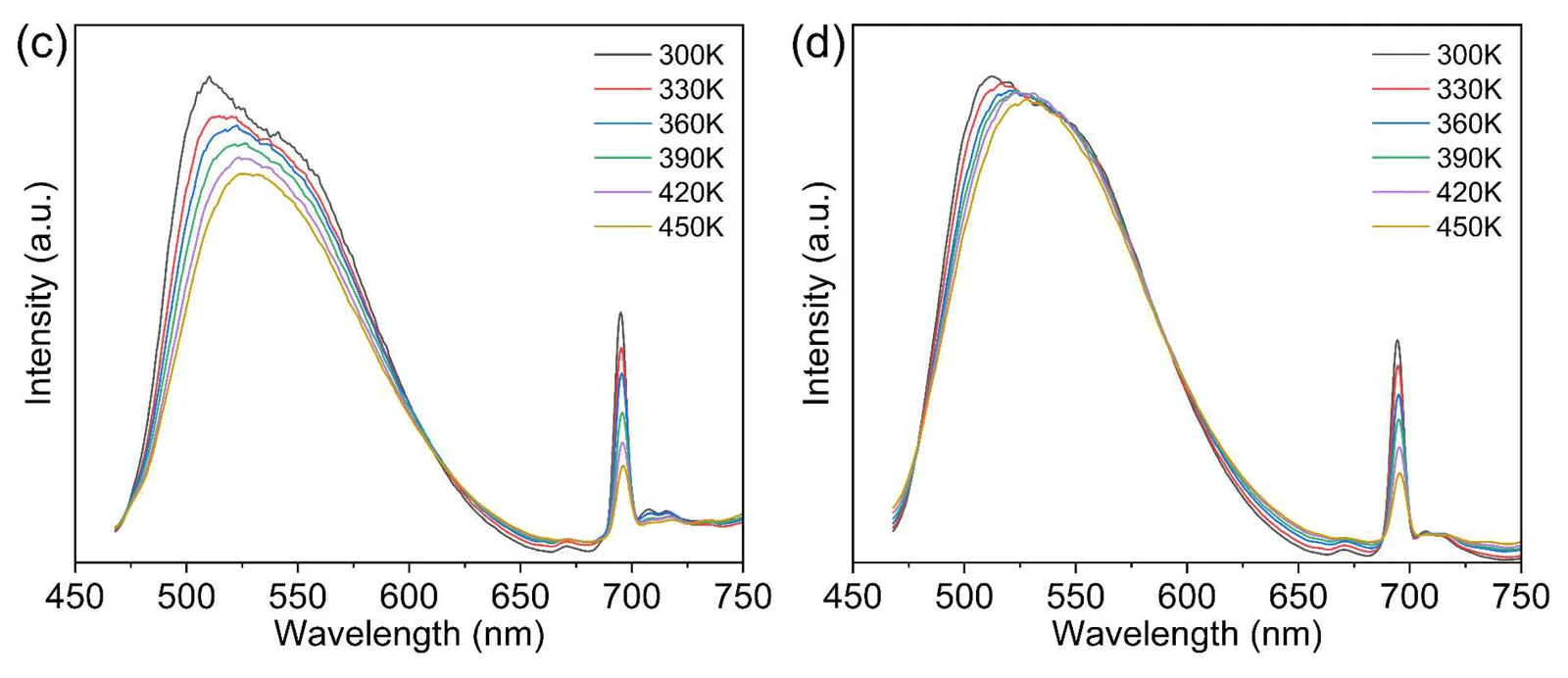
Temperature-dependent PL spectra of Al_2_O_3_-Ce:LuAG CPCs sintered under different temperature–time conditions: (**a**) 1700 °C × 3 h, (**b**) 1725 °C × 3 h, (**c**) 1750 °C × 3 h, and (**d**) 1750 °C × 10 h.

Figure 8 presents the temperature-dependent PL spectra of the Al_2_O_3_-Ce:LuAG CPCs. Under 450 nm blue light excitation, the emission band of the ceramic phosphor appears in the 490–600 nm range, with a peak centered at approximately 510 nm. As the reabsorption of the short-wavelength portion of the emitted light increases with increasing temperature, the emission peak gradually shifts towards longer wavelengths. This phenomenon can be attributed to the augmented electron–phonon coupling within the ceramic. As the temperature increases from RT to 450 K, the PL peak intensities of phosphors sintered at 1700 °C × 3 h, 1725 °C × 3 h, 1750 °C × 3 h, and 1750 °C × 10 h decrease to 61.4%, 78%, 82.1%, and 95.7%. The “1700 °C × 3 h” ceramic sample exhibits an excessively high porosity of 21%, which consequently diminishes its thermal stability. In turn, the excellent thermal quenching behavior of the “1750 °C × 10 h” sample indicates its potential for operation at high excitation power densities.

**Figure 9:**
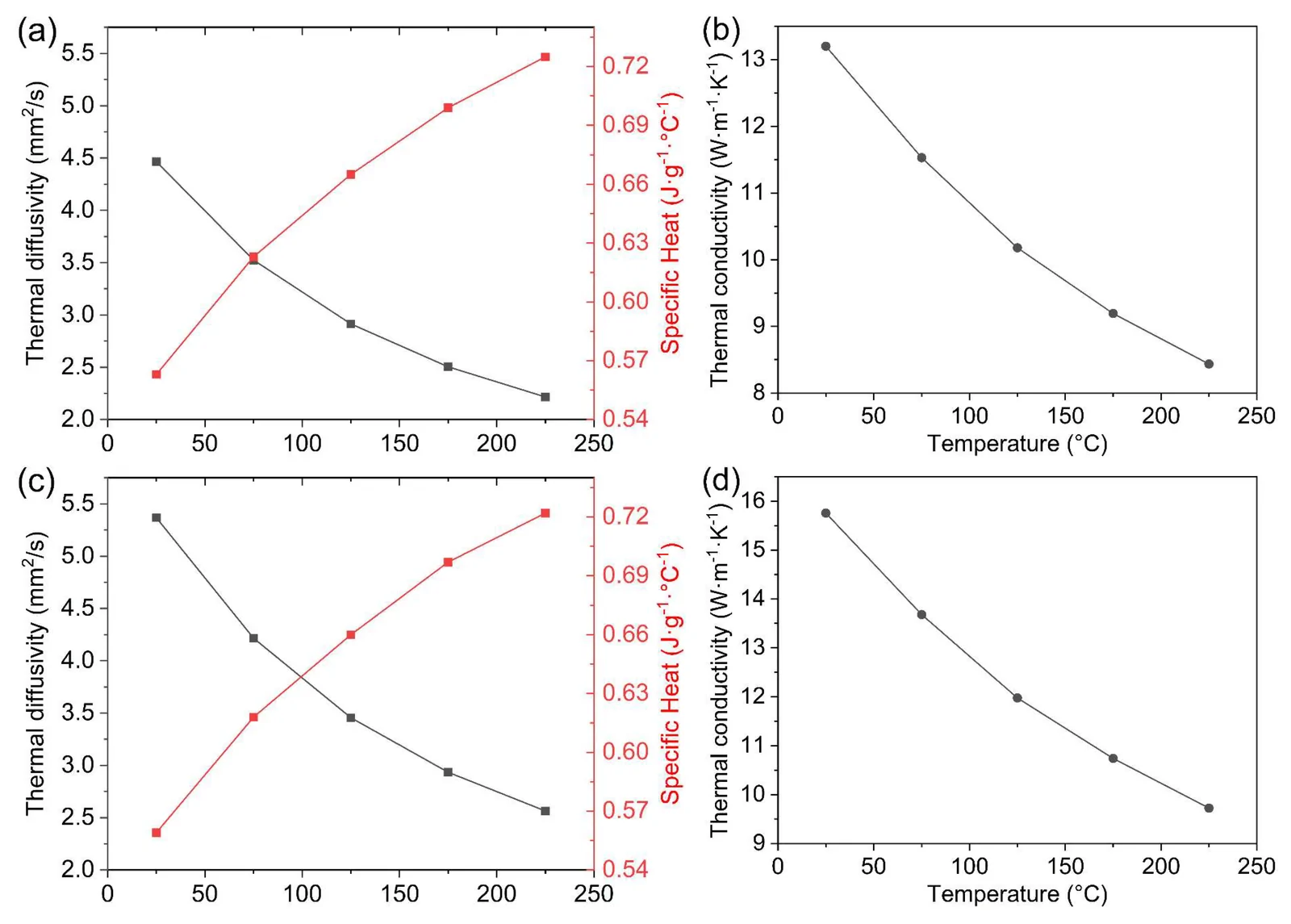
Thermal diffusivity, specific heat capacity, and thermal conductivity as a function of temperature for Al_2_O_3_-Ce:LuAG CPCs vacuum-sintered at (**a**,**b**) 1725 °C × 3 h and (**c**,**d**) 1750 °C × 10 h.

In comparison, Figure 9 presents the temperature-dependent thermal diffusivity, specific heat capacity, and thermal conductivity of Al_2_O_3_-Ce:LuAG CPCs vacuum-sintered at 1725 °C × 3 h and 1750 °C × 10 h. The thermal conductivity typically decreases with increasing temperature, owing to enhanced phonon scattering at the elevated temperatures. As shown in Figure 9d, the thermal conductivity of the “1750 °C × 10 h” sample is 15.6 W·m^−1^·K^−1^ at RT, which is higher than that of monophase Ce:LuAG ceramics (9 W·m^−1^·K^−1^). The thermal robustness of the “1725 °C × 3 h” sample was generally poorer. This phenomenon can be attributed to the fact that, following air annealing, some pores in the ceramic are filled with air. Compared with the LuAG and Al_2_O_3_ phases, air has a significantly lower thermal conductivity of about 0.026 W·m^−1^·K^−1^. Moreover, phonon propagation is the predominant heat-transfer mechanism in PCs. The presence of pores in the ceramic medium acts as scattering centers for phonons, thereby significantly reducing their mean free path. Taken together, these two factors lead to a decrease in the thermal conductivity of the ceramic as its porosity increases.

**Figure 10:**
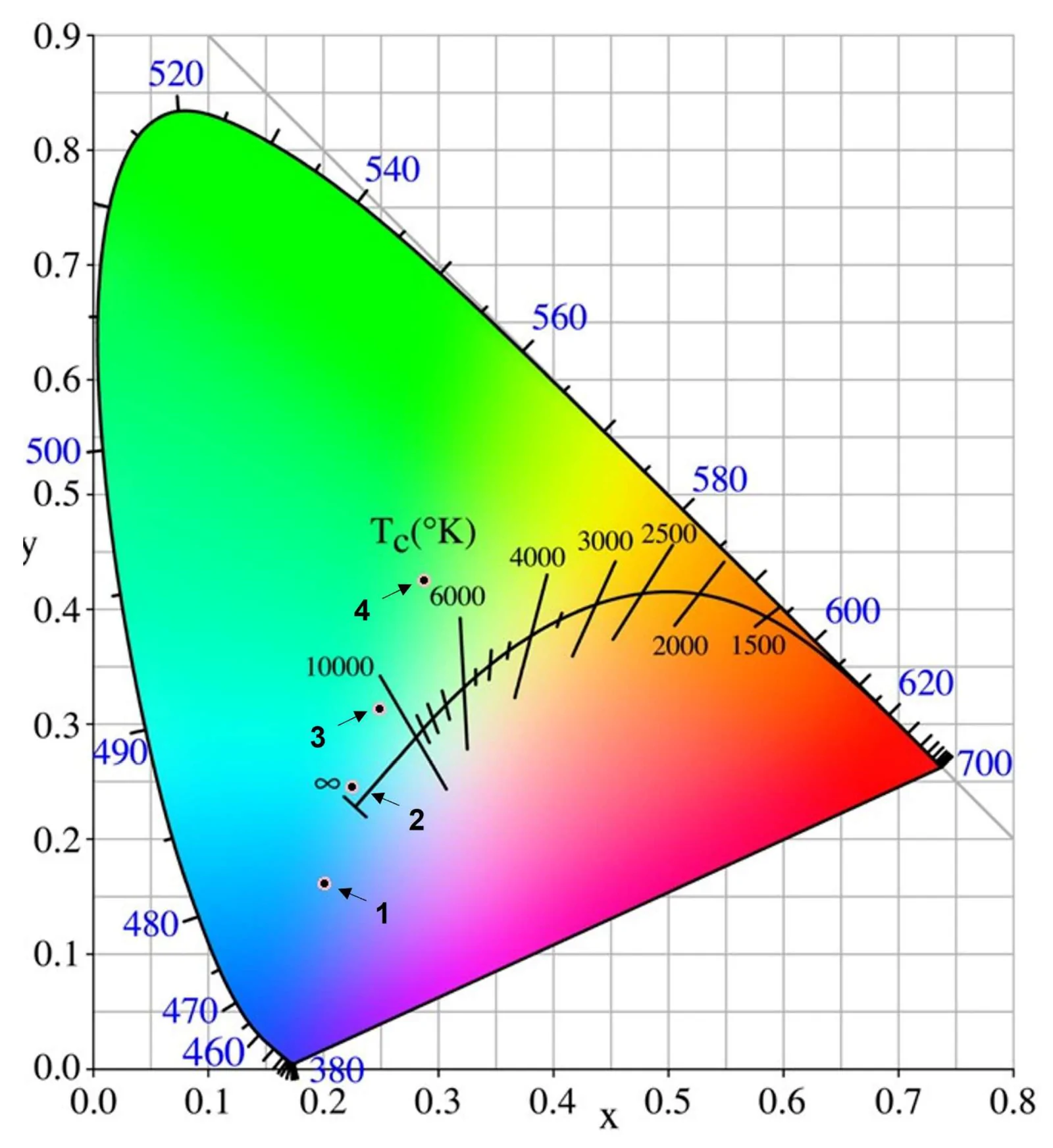
CIE chromaticity coordinates of Al_2_O_3_-Ce:LuAG CPCs under 0.9 W blue laser excitation.

Figure 10 shows the CIE chromaticity coordinates of Al_2_O_3_-Ce:LuAG CPCs under 0.9 W blue laser excitation. With the increase in sintering temperature and holding time, the color coordinates of the CPCs shifted from (0.20, 0.16) in the blue region to (0.29, 0.43) in the yellow-green region.

**Figure 11:**
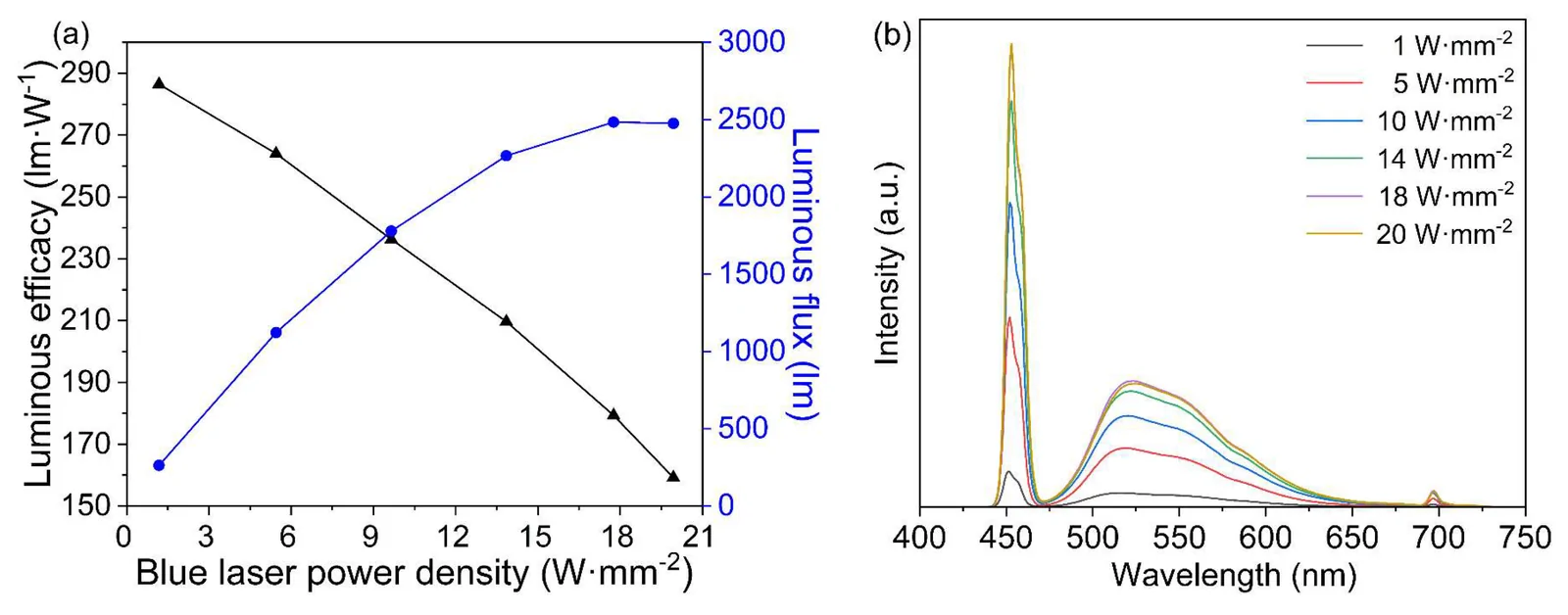
(**a**) LE/ LF values, and (**b**) spectral performance of a 1 mm-thick “1750 °C × 10 h” Al_2_O_3_-Ce:LuAG sample as a function of blue LD excitation power density.

It has been demonstrated that the “1750 °C × 10 h” sample has a maximum LE value of 286 lm·W^−1^ at 0.9 W blue LD excitation. At the same time, the LF increases gradually with increasing incident power density and reaches saturation at 20 W·mm^−2^ (Figure 11a). The emission intensity increases with increasing excitation power, while the luminous intensity remains largely unchanged even under a high incident LD power density of 20 W·mm^−2^ (Figure 11b). These dependencies are consistent with those shown in Figure 6 and Figure 7, indicating that the sample exhibits good thermal performance.

**Figure 12:**
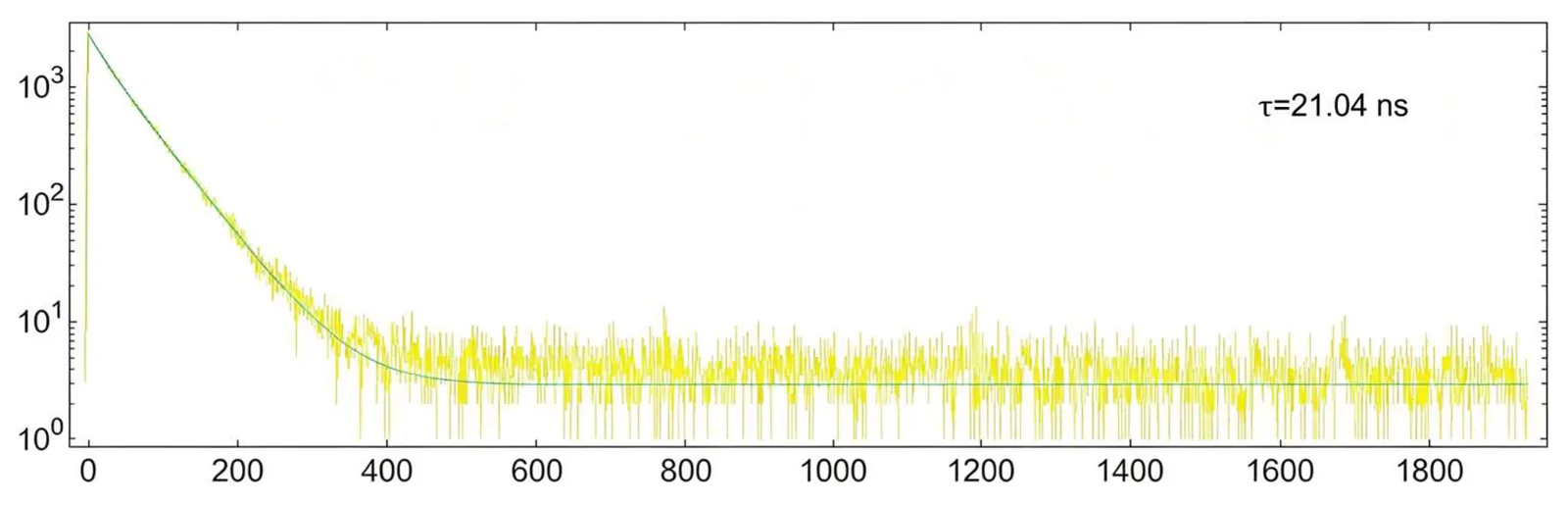
Fluorescence lifetime spectra of Al_2_O_3_-Ce:LuAG CPCs sintered at 1750 °C × 10 h.

The fluorescence lifetime spectra of Al_2_O_3_-Ce:LuAG CPCs sintered at 1750 °C × 10 h is shown in Figure 12. The measured fluorescence lifetime is 21.06 ns. This relatively short fluorescence lifetime is beneficial for increasing the saturation threshold of the ceramic phosphor because a faster decay of the excited state reduces the probability of excited-state absorption and thermal accumulation under high-power excitation, thereby improving the stability and efficiency of the material under high-power laser diode (LD) pumping. This characteristic makes the Al_2_O_3_-Ce:LuAG composite ceramic a promising candidate for high-power, LD-driven lighting applications.

**Figure 13:**
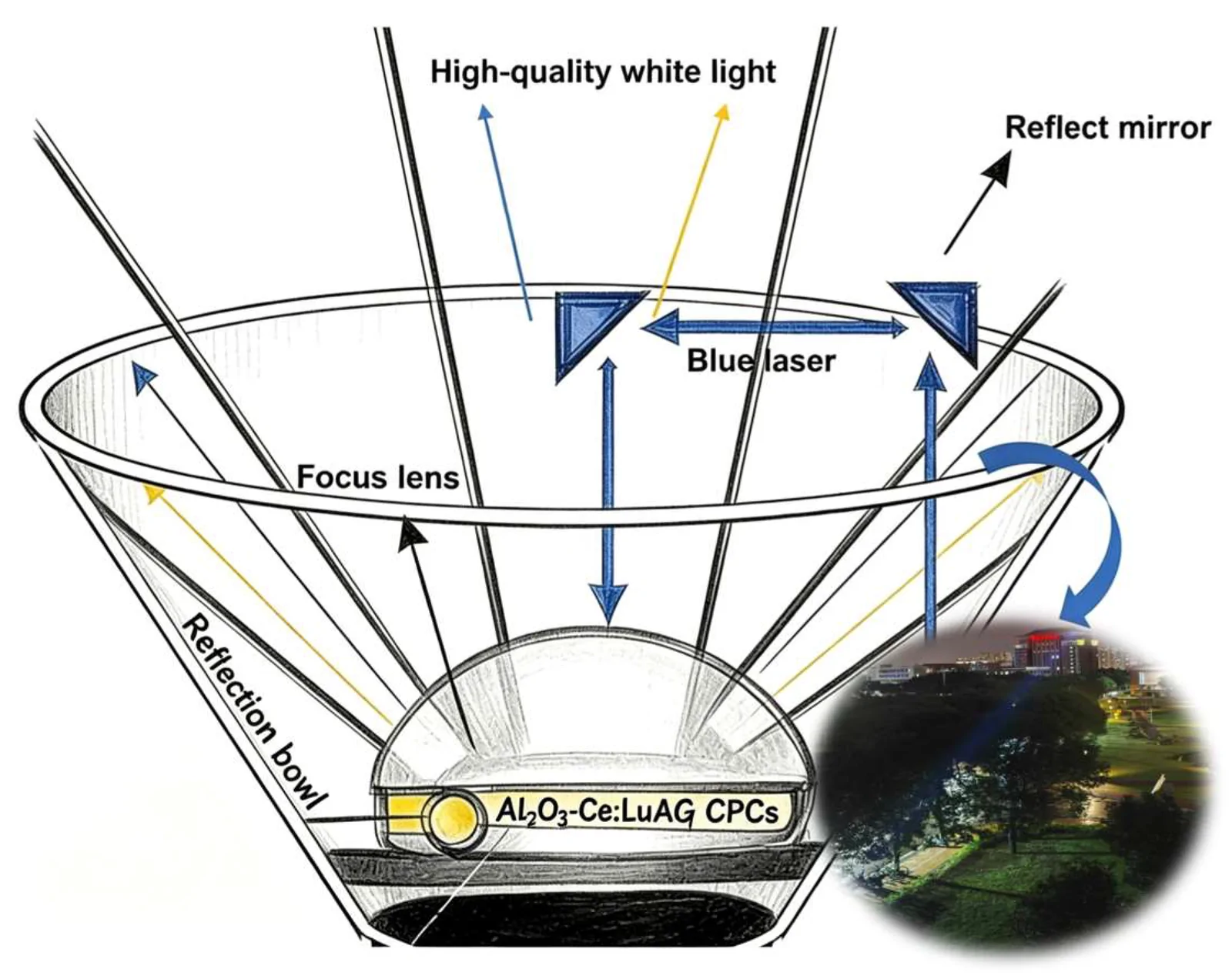
Laser-driven prototype [9].

Figure 13 shows the Illumination diagram for a fluorescence measurement device. The device consists primarily of three components: the reflection bowl, the focusing lens and the reflector. The reflection bowl features an ellipsoidal design that converts the Lambertian emission characteristics of the fluorescent ceramic into directional emission, thereby improving optical efficiency. The focusing lens focuses the laser beam waist onto the surface of the fluorescent ceramic, increasing power density; at the same time, it controls the spot size to prevent excessive energy concentration that could cause localized overheating of the ceramic (thermal quenching, or thermal damage). The reflector folds the optical path, allowing the laser source to be mounted laterally, thereby reducing the overall axial height of the device and optimizing its compactness.

**Figure 14:**
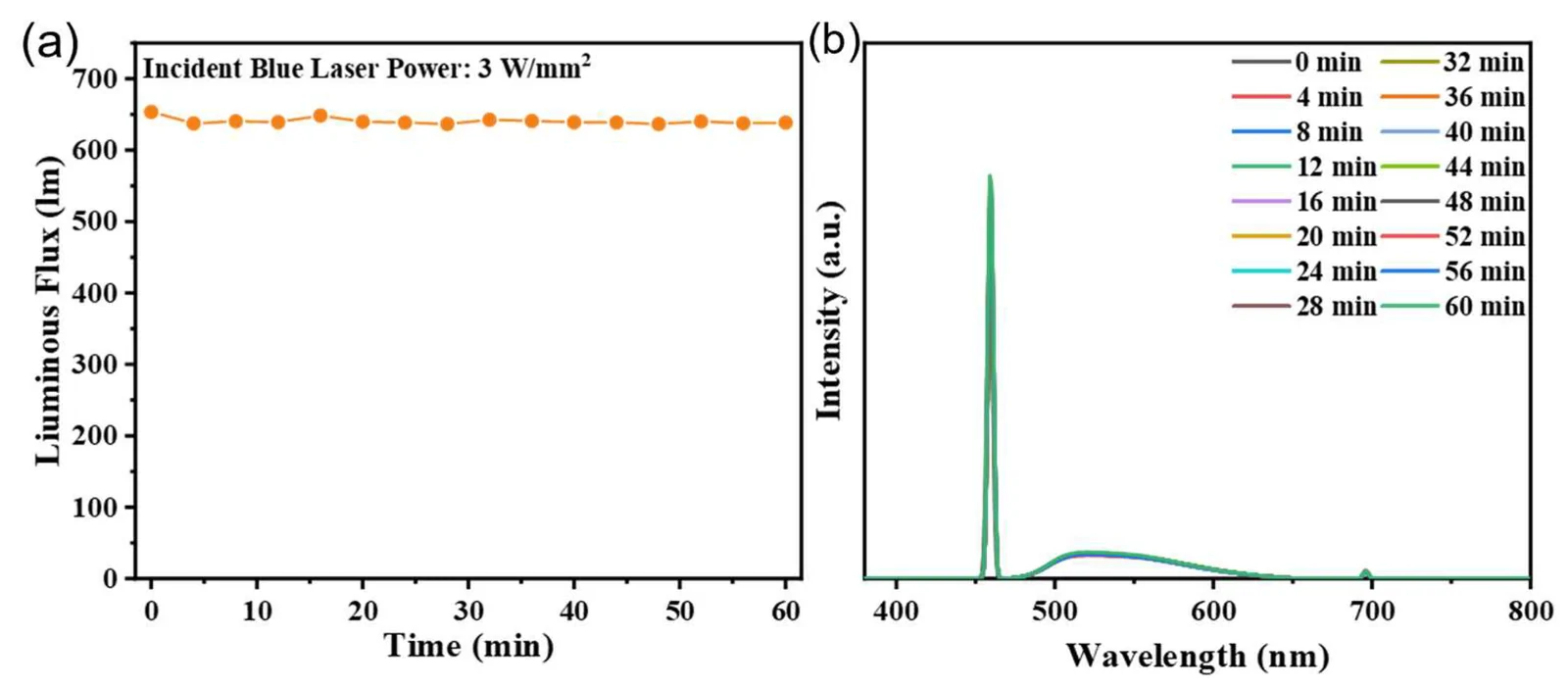
(**a**) LF values, and (**b**) spectral performance of laser-driven prototype lighting source of blue LD excitation power density.

Figure 14 shows the Illumination diagram for a fluorescence measurement device. In the laser-driven prototype lighting source, the blue laser operates at an intensity of approximately 3 W mm^−2^, producing a measured luminous flux of 653.66 lm. Furthermore, after continuous irradiation with the blue laser for 1 h, the luminous flux decreased from 653.66 lm to 638.57 lm, corresponding to a marginal reduction of only ~2.3%.

**Figure 15:**
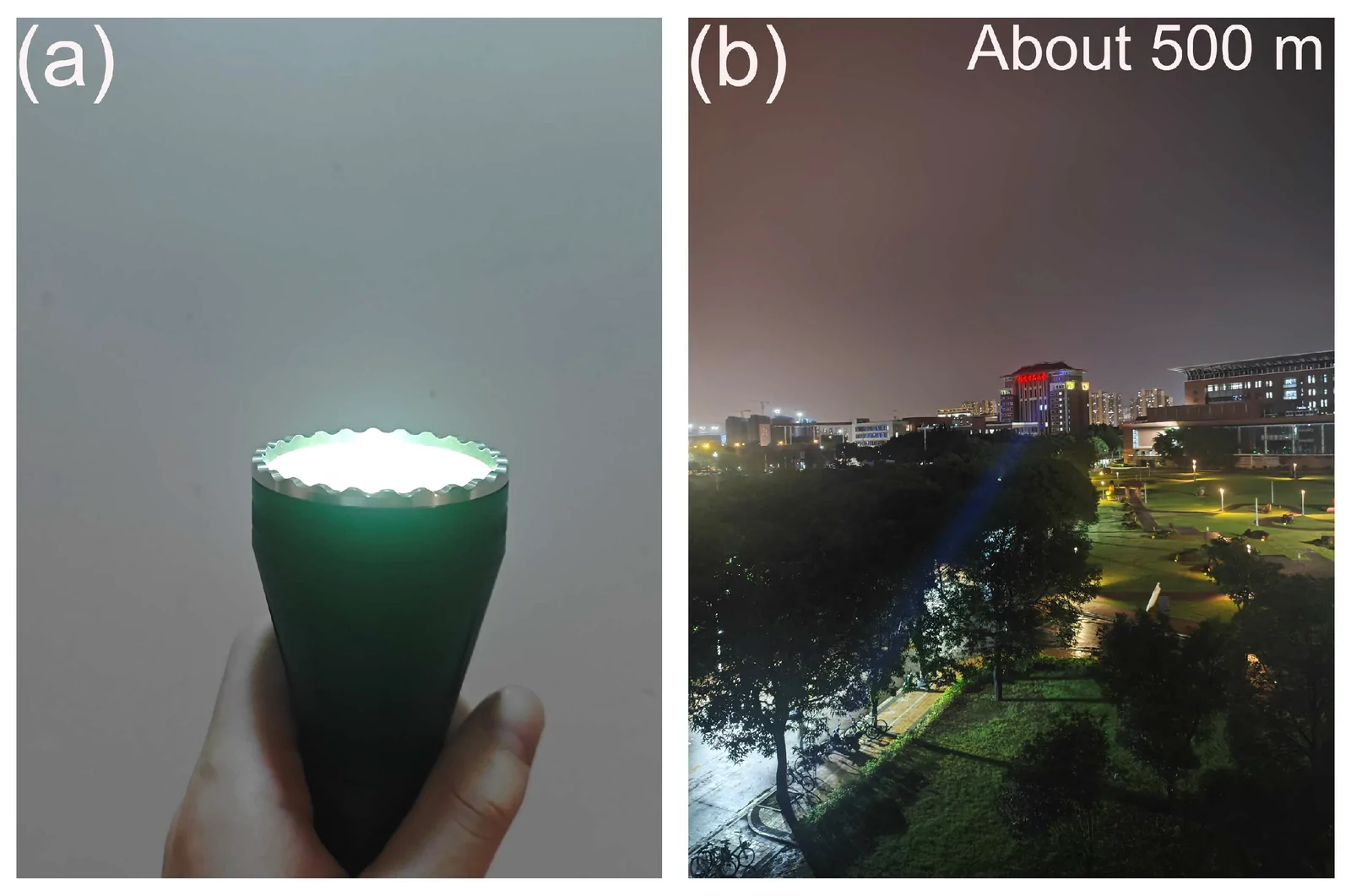
Illumination images of (**a**) LD devices based on Al_2_O_3_–LuAG:Ce CPCs at (**b**) a distance of approximately 500 m.

In order to evaluate the potential of this material for use in lighting applications, a laser-driven lighting device was developed. This device utilized 40 wt.% Al_2_O_3_-0.4at.% Ce:LuAG CPCs in combination with a 10 W blue laser. The prototype lamp emits white light with a range exceeding 500 m. As illustrated in Figure 10, the illumination pattern of the laser-driven lighting device at a distance of 500 m demonstrates its potential for application in next-generation laser-driven lighting technologies, including automotive headlights, military lighting, and outdoor rescue operations (Figure 15).

## 4. Conclusions

In response to the growing demand for green-emitting phosphors for high-power laser lighting, a series of compositionally uniform 40 wt.% Al_2_O_3_-0.4at.% Ce:LuAG PCs was successfully fabricated via vacuum sintering of the corresponding co-precipitated nanopowders. The effects of varying porosity levels, achieved by adjusting the sintering temperature–time conditions, on the microstructure, luminescence and thermal performance of the ceramics were systematically investigated. As the sintering temperature (1700–1750 °C) and holding time (3–10 h) increase, the relative density and thermal stability of the Al_2_O_3_-Ce:LuAG PCs gradually improve. This densification process was accompanied by a continuous decrease in both porosity and average pore size, and statistical grain size distributions of the Al_2_O_3_ and LuAG phases confirmed a uniform biphasic microstructure with moderate grain growth. For the sample with almost no residual pores, sintered at 1750 °C for 10 h and post-annealed at 1450 °C for 10 h, the thermal conductivity at RT was 15.6 W·m^−1^·K^−1^. Concurrently, it exhibited excellent thermal stability, retaining 96% of its PL intensity upon heating to 450 K. The fluorescence lifetime of this vacuum-sintered ceramic was measured to be 21.06 ns, indicating a fast Ce^3+^ 5d→4f decay that is beneficial for suppressing luminescence saturation under intense laser excitation. Under blue LD excitation, the optimized CPC reached a maximum LF of 2500 lm at 20 W·mm^−2^. These results suggest that the designed composite ceramics are suitable for high-brightness laser lighting applications. Finally, the assembly of the ceramic sample with a 10 W blue laser was undertaken to create a laser-driven illumination device. This endeavor resulted in white light output that exceeded 500 m, thereby demonstrating the device’s potential for application in laser-driven lighting technologies.

## Data Availability

The original contributions presented in this study are included in the article. Further inquiries can be directed to the corresponding authors.
